# Intergenerational influences of paternal combat-related trauma on offspring behavioral and brain function

**DOI:** 10.1016/j.ynstr.2025.100767

**Published:** 2025-10-28

**Authors:** Glenn R. Yamakawa, James Freeman, Sydney Harris, Marissa Sgro, Elaina Vlassopoulos, Crystal N. Li, Josep Roman-Juan, Melanie Noel, Sabrina Salberg, Richelle Mychasiuk

**Affiliations:** a Department of Neuroscience, School of Translational Medicine, Monash University, Melbourne, Australia; b School of Medical Sciences, Brain and Mind Centre, University of Sydney, Sydney, Australia; c Department of Psychology, University of Calgary, Canada; d Alberta Children's Hospital Research Institute, Hotchkiss Brain Institute, Owerko Centre, Calgary, Canada

**Keywords:** Allostasis, Chronic pain, Epigenetics, HPA-Axis, Fathers, Military, PTSD

## Abstract

Post-traumatic stress (PTS) is a debilitating mental health condition that is highly prevalent in Veteran populations owing to their increased exposure to combat-related trauma. PTS is associated with numerous comorbid conditions including major depressive disorder, anxiety, and chronic pain. Although the causal mechanisms are currently unknown, paternal trauma has been linked to an increased risk for pathology in their offspring. Therefore, using a preclinical model of combat trauma, we examined the relationship between paternal PTS and offspring socio-emotional functioning, pain perception, and gene expression changes pertinent to HPA-axis functioning, reward processing, and epigenetic regulation. Paternally experienced trauma produced persistent changes in sire anxiety, anhedonia, and sociability, as well as elevated levels of corticosterone and changes to gene expression. In addition, the paternal experiences prior to conception changed offspring behaviour and gene expression but did not modify the offspring's stress response in adolescence. Offspring born to fathers who experienced trauma exhibited changes to nociceptive sensitivity, social and anxiety-like behaviour, as well as changes in expression of *5HT1A*, *5HT2A, Comt, Dnmt3a, Drd2, FKBP5, NR3C1, Maoa*, and *Mecp2* in the adrenal gland, hippocampus, hypothalamus, prefrontal cortex, and thalamus. These results suggest that trauma in fathers may alter expression of genes that contribute to an increased risk for the development of mental health conditions, such as PTS and chronic pain, in their offspring. This model of paternally induced intergenerational transmission could be used to explore the efficacy of future therapeutic strategies to ameliorate some risk imparted upon offspring by Veteran fathers living with PTS.

## 1 Introduction

Post-traumatic stress (PTS) is a debilitating mental health condition characterized by intrusion, avoidance, emotional numbing, and hyperarousal symptoms that often follows a traumatic event (Diagnostic and Statistical Manual of, 2013). It has a prevalence rate of 11.1 % in civilians, however this increases to 24.5 % in Veteran populations owing to their increased exposure to combat-related trauma (Spottswood et al., 2017). PTS is associated with a multitude of comorbid conditions, such as major depressive disorder, anxiety disorders, suicidal ideation, insomnia, substance abuse, and chronic pain (Debell et al., 2014; Hall Brown et al., 2015; Scioli-Salter et al., 2015), with prevalence rates of comorbid mental and physical health conditions in Veterans with PTS estimated to be as high as 66 % (Shipherd et al., 2007). Research indicates that PTS may be driven, at least in part, by dysregulation and over-activation of stress and fear/threat processes which are characterized by altered functioning within the hypothalamic-pituitary-adrenal (HPA) axis, the amygdala-hippocampus-prefrontal cortex circuit, neurotransmitter systems, and neuroinflammatory processes (Scioli-Salter et al., 2015; Ressler et al., 2022; Freeman et al., 2025).

Literature related to civilian populations has also linked extreme stress and PTS to increased pathology in subsequent generations (Yehuda et al., 1998, 2005), with studies specifically demonstrating that children born to Holocaust survivors who developed PTS, exhibited greater vulnerability to PTS and PTS-related symptoms, than children born to parents without a family history of war exposure (Yehuda et al., 1998; Solomon et al., 1988). As these stress and trauma exposures often predate conception, epigenetics may be one of the driving mechanisms behind this intergenerational transmission, whereby environmental conditions influence gene expression, in a heritable manner (Rampp et al., 2014; Sheerin et al., 2017; Hjort et al., 2021). Briefly, experiences such as trauma can lead to DNA methylation, histone modification, and RNA regulation which subsequently alter gene expression and individual phenotypes (Freeman et al., 2024; Jaenisch and Bird, 2003; Raza et al., 2023; Mohn and Schübeler, 2009). Ample studies have demonstrated maternal transmission of these risks, whereby prenatal and neonatal conditions influence offspring outcomes (Champagne, 2010). Although the literature on paternal influences is more limited, growing evidence supports similar findings (Mychasiuk et al., 2013; Chan et al., 2018; Eyolfson et al., 2021). Given that spermatogenesis occurs across the lifespan, unlike oogenesis (Hehar and Mychasiuk, 2015a), paternal experiences have an even greater potential to alter gene expression in the next generation (Hehar et al., 2017; Hehar and Mychasiuk, 2015b).

When offspring outcomes are negatively influenced by parental experiences, the pathological manifestations often emerge during adolescence (Schlotz and Phillips, 2009; Van De Berg et al., 2008). Exaggerated or abnormal responses to an already rapidly changing adolescent brain have been linked to peaks in mental health disorders that emerge during this period (Paus et al., 2008; Kessler et al., 2005; King et al., 2011). Therefore, experiences such as psychological stressors during this critical window of development and plasticity can have lasting effects (Welberg and Seckl, 2001). This is particularly relevant given that parental trauma has been linked to increased family conflict and altered parenting behaviours (Freeman et al., 2024; Lehrner et al., 2014), both of which also interact with adolescent development to modify susceptibility to long-term mental and physical health conditions.

Therefore, we used a preclinical model of combat-related trauma to examine the intergenerational effects of paternal trauma on offspring outcomes. More specifically, we investigated the relationship between paternal trauma exposure prior to conception and offspring socio-emotional functioning, pain perception, and gene expression changes pertinent to HPA-axis functioning, reward processing, and epigenetic regulation (*5HT1A, 5HT2A, Comt, Dnmt3a, Drd2, FKBP5, NR3C1, Maoa,* and *Mecp2*). We hypothesised that offspring born to fathers who experienced trauma would exhibit increased nociceptive sensitivity and socio-emotional impairments, along with altered expression of genes important for stress reactivity, neurotransmission, and DNA methylation. To investigate this intergenerational relationship, Sprague Dawley rat sires were assigned to a combat trauma or a control condition, before being mated with control dams. Offspring were then exposed to a stressor in adolescence, followed by behavioural testing. Tissue was collected from sires and their offspring to examine gene expression changes in the adrenal glands, hippocampus, hypothalamus, prefrontal cortex (PFC), and thalamus.

## 2 Materials and methods

### 2.1 Animals

All experiments were approved and completed in accordance with the Alfred Medical Research and Educational Precinct (AMREP) Animal Ethics Committee (E/8419/2023/M). Power calculations were completed in G∗Power (version 3.1) using Cohen's (1988) criteria (Cohen, 1998), (effect size = 0.45, alpha = 0.05, and power = 0.80). Given our primary analyses required three-way ANOVAs, sample sizes of 79 was adequate. Our experimental sample size was 90 offspring. Each group contained ∼11 animals and are identified with individual data points in all offspring graphs. For control sires, 2–3 offspring per sire were used for each group and for combat trauma sires 1–2 offspring per sire were used for each group. Statistical analyses were conducted prior to all offspring behavioural and gene expression analyses to ensure that there were no effects of sire or litter. The animal vivarium was maintained on a 12-h light/dark cycle with lights on at 0700 and temperature maintained at 23 °C.

### 2.2 Sire combat trauma paradigm

Adult male Sprague Dawley rats were obtained from the Monash Animal Research Platform at approximately postnatal day (p) 56. Sires were pair-housed and had access to food and water *ad libitum*. Sires were randomly assigned to either the control group (n = 4) or the combat-related trauma group (n = 8). Sires in the control group remained pair housed throughout the experiment. Over the 3-week stress period, control sires were removed from their home-cage each morning and weighed but were not exposed to the stress protocols. Rats in the combat trauma group underwent 3-weeks of random stress designed to mimic combat trauma beginning on p65. This involved one stressor per day, 5 days/week, during the light cycle (between 0700h and 1900h). Throughout the week (Monday to Friday), while undergoing the stressors, the sires in the combat trauma group were also socially isolated as they were housed individually, being returned to their cage mates for 48 h (Saturday and Sunday). No aggression was observed following re-introduction of sires to their cage mates on any of the occasions. The five stressors included predator exposure stress (PES), predator scent stress (PSS), acoustic stress, restraint stress, and a mild traumatic brain injury (mTBI), all of which are detailed below. At the end of the 3-week stressor paradigm, rats from the combat trauma group were permanently re-paired with their cage mate; cage mates underwent the same paradigm. This combat-trauma protocol was designed to expose sires to stressors with greater face validity for Veterans, encompassing moderate-to-severe stress protocols involving visual, auditory, olfactory, and tactile stimuli as well as a mTBI/concussion.

1. *Predator Exposure Stress:* Combat trauma sires were subjected to one 20 min hawk exposure each week, over the 3-week period (3 sessions total). The date of the hawk exposure was randomly assigned each week. Sires were placed in a 50 x 50 × 50cm plexiglass box with a plexiglass lid containing small air holes. They were habituated to the enclosure for 5 min, following which two model hawks (40 cm high x 15 cm wide x 15 cm long) were placed in close proximity to the plexiglass box for 20 min; one hawk was placed next to the plexiglass box in clear view for the rat while the other hawk was hung from the ceiling above the plexiglass box. Exposure to model hawks was conducted to induced stress via the visual system (Mulungu and Martin, 2024; Yang et al., 2020). The plexiglass box was cleaned with 80 % ethanol between each animal.
2. *Predator Scent Stress:* Sires in the combat trauma group underwent one 10-min exposure to fox urine (Muroy et al., 2016), each week, over the 3 week stress paradigm (3 sessions total). The date of the scent exposure was randomly assigned each week. Sires were transported from their home cage to the stress exposure room in a separate cage to ensure the fox urine scent was not transferred to vivarium. In the stress exposure room, sires were habituated to a 50x50 × 50cm plexiglass box for 2 min. On completion of the habituation phase, a petri dish containing a small piece of gauze soaked in 1 ml of Red Fox urine (Wildlife Research Center, MN, USA) was placed in the centre of the plexiglass box with the sire for 10-min. Following the 10-min stressor phase, the sire was removed from the box and returned to the transport cage for transport back to their home cage in the vivarium. The plexiglass box was cleaned with 80 % ethanol between each animal. Exposure to the fox urine was designed to induce stress via the olfactory system.
3. *Acoustic Stress:* Sires in the combat trauma group were exposed to one acoustic stress session each week, for 3-weeks (3 sessions total) (Miller et al., 2021). The date of the acoustic stress exposure was randomly assigned each week. The startle pre-pulse inhibition (PPI) for the rat single cage system by Ugo Basile was used (APAC Scientific). Sires were placed in the animal holder within the isolation cubicle to habituate for 5 min. Following the habituation period, each animal was subjected to 15 startle pulses lasting 40 ms at 100 dB, 105 dB, 110 dB, 115 dB, and 120 dB; 15 white noise pulses lasting 40 ms at 100 dB, 105 dB, 110 dB, 115 dB, and 120 dB; and 15 pulses with white noise pulses at 100 dB, 105 dB, 110 dB, 115 dB, and 120 dB. Background noise was set at 65 dB for the entire experiment and all acoustic stimuli were programmed to be delivered in a random order. The animal holder of the acoustic startle apparatus was cleaned with 80 % ethanol between each animal. Exposure to acoustic stimulus was implemented to induce stress via the auditory system.
4. *Restraint Stress:* Sires in the combat trauma group were exposed to restraint stress on one day of each week, for 3-weeks (3 sessions total) (Donohue et al., 2006). The date of the restraint stress was randomly assigned each week. Each sire was individually placed into a restraint tube which was subsequently placed into a fresh cage for the duration of the stress protocol. Sires were immobilised in the restraint tube for 1-h. Restraint tubes were thoroughly cleaned with 80 % ethanol between each animal. Exposure to the restraint tube was used to induce physical stress via the tactile/proprioception system.
5. *Mild Traumatic Brain Injury (mTBI) and Blood Collection:* All rats received either a blood collection (control group) or mTBI + blood collection (combat trauma group) once per week, over the 3-week period (3 total). Blood was collected each week, for corticosterone analyses. Animals were anesthetised with 5 % isoflurane at 1L/min O_2_ until non-responsive to a toe-pinch (∼90 s) then transferred to a nose cone at 2–2.5 % isoflurane at 1L/min O_2_. Rats were placed in a prone position with their tail overhanging the edge of the bench, which was submerged in warm water before being disinfected with chlorohexidine. A 27G needle was used to collect blood from a lateral tail vein in serum separator tubes, which was clotted at room temperature for 30 min. Tubes were then centrifuged at 6000*g* at 4 ° C for 1 min, serum aliquoted, and stored at −80° C for downstream ELISA processing of corticosterone levels (Invitrogen, USA). Following blood collection, control rats were placed in the lateral impact device but did undergo a mTBI. They were immediately returned to a clean, warm cage and monitored until full recovery before being returned to their home cage. For the mTBI, combat trauma sires were removed from the nose cone and immediately placed in a prone position on a Teflon board with the left side of their head adjacent to an aluminium “helmet” to prevent skull/bone damage. The mTBI was induced via the lateral impact method, whereby a 50g weight was propelled toward the rat's head at a mean of 10.03 ± 0.21 m/s. Upon impact, the sires underwent a 180° rotation that results in acceleration/deceleration and rotational forces being imparted upon the brain. Following the mTBI induction, combat trauma sires were placed in a fresh cage on a heat pad in the supine position. The time it took the sire switch to a prone position was recorded as the time-to-right and has been used as a measure of loss of consciousness. A significant proportion of concussive injuries sustained in combat are non-blast related (Wilk et al., 2010), with research demonstrating that PTSD severity and neuropsychological impairments were indistinguishable for US military personnel who sustained blast and non-blast related concussions (Mac et al., 2014). Veterans who experienced brain injuries during active duty are also more likely to experience chronic pain and mental health disorders (Karr et al., 2025)

### 2.3 Sire mating and behavioural testing

**Fig. 1:**
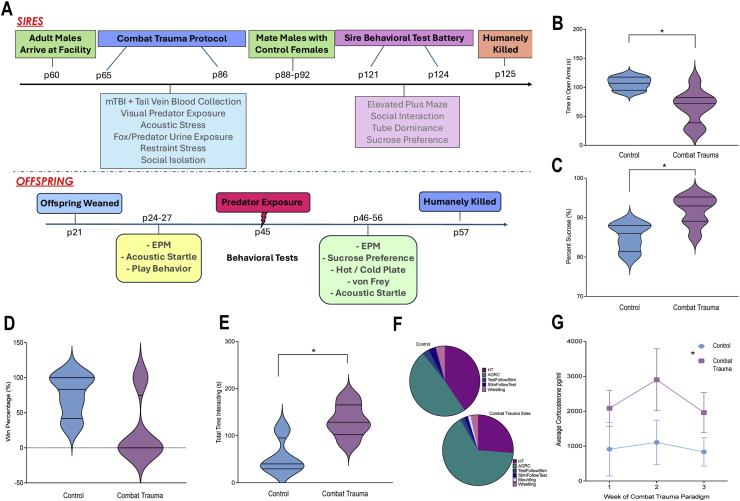
(A) Experimental timeline for sires and offspring, p – post-natal day. Violin plots and pie charts displaying behaviour and corticosterone results for the sires. Significant effect of sire group indicated by ∗; *p's* < 0.05. B) Time spent in the open arms of the elevated plus maze; C) Percent sucrose consumed for the sucrose preference test; D) Win percentage in the tube dominance task; E) Total interaction time in the social interaction test; F) The distribution of specific behaviours identified in the social interaction test; and G) Corticosterone levels across timepoints. HT - head torso sniffing, AGRC - anogenital reciprocal circle.

Two days following the completion of the combat trauma paradigm, each sire was paired with a distinct control female (p86) for four days, to ensure that mating encompassed all 4 days of the female's estrous cycle. Following the four days of mating, sires were rehoused with their original male cage mate for the duration of the experiment, having no contact with their subsequent offspring. There was no aggression between male cage mates upon reintegration. Following mating, females were also pair housed until gestational day (G) 19 at which point they were individually housed for nesting and birth, where they remained individually housed with their litters until offspring weaning at p21. Thirty days following the completion of mating, sires underwent a battery of tests to examine behavioural outcomes associated with combat trauma. Beginning on p121, sires were tested for anxiety-like behaviour with the elevated plus maze (EPM) (Walf and Frye, 2007), social deficits with the social interaction task (Pellis and Pellis, 1990), aggressive behaviours in the tube dominance task (Lindzey et al., 1961), and anhedonia with the sucrose preference task (Primo et al., 2023). See Fig. 1A for experimental timeline.

*EPM:* Briefly, rats were habituated to the testing room for 30 min and were subsequently placed in the center of a raised (60 cm) plus shaped maze, consisting of two closed and two open arms. All testing occurred between 0900 and 1400 under indirect white light (480 lux). During a 5-min trial, the rat was placed in the centre of the maze and time spent in each of the arms was measured by overhead TopScan Lite Software (Clever Sys Inc., Reston, VA), tracking software, with more time spent in open arms indicating decreased anxiety.

*Social interaction:* Animals undergoing the social interaction test were single-housed the day prior to testing. Rats were placed in a clear plexiglass box (50 cm^3^) with an age, weight, and sex matched stimulus rat, under red light conditions. The animals were allowed to freely interact for 7 min, with the session being video recorded and later scored by a researcher blinded to experimental conditions for a variety of play parameters.

*Tube dominance:* Each of the sires was weight-matched to a naïve stimulus rat and they were simultaneously released at either end of a clear, narrow tube. The animals were allowed to interact in the middle of the tube, with the more dominant animal forcing the other out of the tube. When one animal had all four paws outside the tube, the test was considered complete. Each pair of rats underwent this procedure 3 times each, with each round being limited to 3 min. All testing occurred between 0900 and 1100. The plexiglass tube was cleaned with 80 % ethanol between each round.

*Sucrose preference:* At the start of this paradigm, rats were singly housed, in their home cage were presented with one bottle filled with a 1 % sucrose water solution, and the other with normal water. These bottles were weighed beforehand, and again after a 24-h period of *ad libitum* access. Sucrose preference was calculated as a percentage of the volume of sucrose intake over the total volume of fluid intake. The greater percentage of sucrose intake, the lesser the anhedonia-like phenotype.

*Acoustic startle:* The PPI system, as described in the acoustic stress paradigm was used. At the beginning of the experiment, each rat was placed in the rat holder and habituated to the acoustic startle box for 5 min without any auditory stimuli. Following the initial habituation period, rats were given three blocks of random startle sounds. The first block was for startle pulse habituation, the second block for pre-pulse inhibition testing, and the third block was for acoustic startle response testing. Background noise was set at 65 dB for the entirety of the experiment. Block one consisted of five 120 dB startle pulses lasting 40ms without pre-pulses with randomly variable inter-trial intervals ranging between 10 and 20 s. Block two consisted of pre-pulses (0, 69, 73, 77, and 81 dB) that lasted 20ms. The time interval between each pre-pulse and pulse tone was set at 100ms. Rats were exposed to each pre-pulse 5 times in random order for a total of 25 trials. After each pre-pulse tone, rats received a 120 dB startle pulse alone tone of 40ms duration and the rats' startle response was recorded. Inter-trial time intervals for the 25 trials in block two were randomly variable ranging from 10 to 20 s. Rats received 5 exposures of different pulses in random order. The peak-to-peak amplitude of the animals' startle responses was recorded in each trial and all experiments were conducted during the rats’ light cycle. The animal holder was cleaned with 80 % ethanol between each rat.

*ELISA – Corticosterone:* Serum collected during the 3 mTBI procedures was used for corticosterone analyses. Serum samples and reagents from the corticosterone competitive ELISA kit (Invitrogen, USA) were brought to room temperature prior to use. To prepare the serum samples, 5 μL of serum was mixed with 5 μL of the provided dissociation reagent and incubated at room temperature for 5 min. Following incubation, the samples were diluted 1:100 with 1X assay buffer. Standards were prepared starting at 10,000 pg/mL corticosterone and serially diluted 1:2 to generate eight standards ranging from 5000–39.063 pg/mL. A blank (0 pg/mL corticosterone) and a non-specific binding control were also included. 50 μL of standards and samples were pipetted in duplicate into the provided 96-well plate. Absorbance was measured within 10 min using a microplate reader (Thermo Scientific Multiskan GO) at 450 nm. A standard curve was generated from 5000–39.063 pg/mL, and background absorbance for all standards and samples was corrected using the non-specific binding control. To determine sample concentrations, optical densities were multiplied by the initial dilution factor, 100, to correct for the sample dilution.

### 2.4 Offspring

When offspring were weaned (p21), they were housed in same-sex, same-condition, groups of 3–4. At p25 they began behaviour testing, which included EPM, social play/interaction and hypervigilance via an acoustic startle paradigm. At p45, half the pups underwent an adolescent challenge via the predator exposure stressor as described for the sires. Following this, at p46 all pups underwent a full battery of behaviour tests, including EPM, sucrose preference, acoustic startle, as described above, as well as the hot/cold plate (thermal nociception), and von Frey (mechanical nociception) tasks (Le Bars et al., 2001; Chaplan et al., 1994).

*Hot Cold Plate:* The apparatus consisted of a temperature controlled circular plate, enclosed with a plexiglass cylinder. For 2 days prior to testing, rats were habituated to the apparatus by being placed on the room-temperature plate for 2 min each day. On the third day, the plate was turned on to hot (52 ° C), and the animals placed inside, with latency to react indicated by hind paw withdrawal being closely monitored. Rats were immediately removed from the plate once a response was observed. They were placed back into their home cage for >1 h before repeating the procedure at the cold setting (2° C). For the cold setting, 2 min was set as the cut off time to remove animals if they did not react. The longer the latency, the greater the thermal nociceptive threshold (Yalcin et al., 2009).

*Von Frey:* For the 2 days prior to testing, rats were placed in boxes on top of a small wire grid and allowed to habituate for 20 min each day. On the third day, rats were again habituated for 20 min, before increasing size filament weights (force, log_10_) were applied: 1.65 g (8 mg, 0.903); 2.36 g (20 mg, 1.301); 2.44 g (40 mg, 1.602), 2.83 g (70 mg, 1.845), and 3.22 g (160 mg, 2.204), 5x to each hind paw. Once the rat displayed a 5/5 reaction the test was stopped, and this filament weight was recorded. The larger the filament weight, the greater the mechanical nociceptive threshold (Deuis et al., 2017).

### 2.5 Euthanasia & RT-qPCR

On p125 for sires and p57 for offspring, animals were weighed and humanely killed for sample collection via isoflurane inhalation followed by rapid decapitation. Brains were weighed and dissected, with the hippocampus, hypothalamus, thalamus, and PFC, flash frozen and stored at −80 ° C for downstream gene expression analysis. Ear tissue and adrenal glands were collected, flash frozen, and stored at −80° C for downstream telomere length and gene expression analysis, respectively. Ear notch tissue was used for telomere length analyses as we have previously demonstrated that changes here are representative of telomere length changes in the brain (Hehar and Mychasiuk, 2016a).

DNA was extracted from ear notch tissue according to the DNeasy Blood and Tissue Kit (Qiagen). DNA concentration and purity was measured using the QIAxpert (Qiagen), then diluted to 30ng/ μ l. Telomere length was analysed via RT-qPCR on the QuantStudio7 system (Qiagen) with all samples run in duplicate using 1 X SYBR Green FastMix ROX, as previously described (Mychasiuk et al., 2015; Hehar and Mychasiuk, 2016b). To determine telomere length, the telomere to single copy ratio (T/S) was calculated and a linear regression equation, established by Cawthorn (2002), y = 1910.5x+ 4157 (where y = telomere length and x = −2^−ΔCt^) was then used to determine relative telomere length.

RNA was extracted from adrenal and brain tissue using the RNeasy Mini Kit (Qiagen) in conjunction with the QIAcube (Qiagen), according to manufacturer's protocols. Concentration and quality were measured with the QIAxpert (Qiagen), before 2 μg of RNA were reverse transcribed to complementary DNA (cDNA) using qScript™ XLT cDNA SuperMix (Quantabio). cDNA was used for RT-qPCR, run on the QuantStudio7 system (Qiagen). All samples were run in duplicate on a 384-well plate, normalized to the housekeeping genes *Cyca* and *Ywhaz*. Each well contained 20 ng cDNA, 1 X SYBR Green FastMix ROX, and 0.5 μM of forward and reverse primers, with the 2^−ΔΔCt^ method used for analysis (Pfaffl, 2001). All primers were obtained from IDT, with a Tm of 60 ° C for all genes, except *Tel* (56 ° C) and *36B4* (54.8 ° C), and primer sequences and cycling parameters shown in Table 1.

**Table 1:** Primer sequences and cycling parameters for RT-qPCR.

Gene symbol	Gene name	Primer sequence	Cycling parameters
*CYCA*	Cyclophilin A	(+) agcactggggagaaaggatt (−) agccactcagtcttggcagt	1 cycle 95 °C 20 sec 40 cycles 95 °C 1 s 40 cycles Tm°C 20 s + Melt Curve
*YWHAZ*	Tyrosine 3-monooxygenase	(+) ttgagcagaagacggaaggt (−) gaagcattggggatcaagaa	
*5HT1A*	Serotonin Receptor 1A	(+) ccgcacgcttccgaatcc (−) tgtccgttcaggctcttcttg	
*5HT2A*	Serotonin Receptor 2A	(+) aacggtccatccacagag (−) aacaggaagaacacgatgc	
*AVP*	Arginine vasopressin	(+) tgctacttccagaactgccc (−) aggtagttctcctcctggc	
*COMT*	Catechol-O-methyltransferase	(+) atcttcacggggtttcagtg (−) gagctgctggggacagtaag	
*DNMT3a*	DNA Methyltransferase 3a	(+) gggtgctatctctctttgatgg (−) ctggatatgcttctgtgtgacg	
*Drd2*	Dopamine receptor 2	(+) gccgagttactgtcatgattgc (−) ggcacgtagaatgagacaatgg	
*FKBP5*	FK506 binding protein prolyl isomerase 5	(+) gaacccaatgctgagcttatg (−) atgtacttgcctcccttgaag	
*NR3C1*	Glucocorticoid receptor	(+) agcttcaggatgtcattacggg (−) gagcttcaaggttcattccagc	
*MAOA*	Monoamine oxidase A	(+) gccaggaacggaaatttgtagg (−) ttggtttctctcaggtggaagc	
*MECP2*	Methyl-CpG Binding Protein	(+) cgtccccttgcctgaaggttgga (−) ctttccagcagagcgaccag	
*36B4*	Acidic ribosomal phosphoprotein P0	(+)cagcaagtgggaaggtgtaatcc (−)cccattctatcatcaacgggtacaa	1 cycle 95 °C 3 min 30 cycles 95 °C 15 s 30 cycles Tm°C 1 min + Melt Curve
*Tel*	Telomere	(+)ggtttttgagggtgagggtgagggtgagggtgagggt (−)tcccgactatccctatccctatccctatccctatcccta	1 cycle 95 °C 3 min 30 cycles 95 °C 15 s 30 cycles Tm°C 2 min + Melt Curve

### 2.6 Statistics

SPSS v28.0 for Mac was used for statistical analysis, with a p value of <0.05 considered significant. For sire data, Mann-Whitney tests were run to compare behaviour, weight data, and gene expression between control sires and combat trauma sires. A one-way repeated measures ANOVA was run for sire corticosterone levels. For the offspring behaviour data that was collected prior to the adolescent challenge, two-way ANOVAs were run with sex (male, female) and paternal group (control, combat trauma) as factors. For behavioural data collected following the adolescent challenge, three-way ANOVAs were run with sex (male, female), paternal group (control, combat trauma), and adolescent challenge (control condition, adolescent stressor) as factors. Post-hoc Bonferroni pairwise comparisons were run, where applicable, for significant interactions.

## 3 Results

### 3.1 Sire behavior

No significant differences were found in sire body weight, brain weight, or spleen weight in response to the paternal combat trauma exposure, *p's* > 0.05. In addition, there were no significant differences in dam weight gain, litter size, or ratio of male to female offspring born for dams mated with control or paternal combat trauma sires, *p's* > 0.05 (data not shown).

Mann-Whitney U tests were performed to compare behaviors for sires from the control and combat trauma groups, see Table 2 for statistical results. Combat trauma sires spent significantly less time in the open arms (Fig. 1B) and consumed a higher percent sucrose (Fig. 1C). There was a trend towards significance in the win percentage for the tube dominance task (Fig. 1D), and combat-related trauma sires spent more time interacting with a novel rat, than control sires (Fig. 1E). See Fig. 1F for distribution of behaviors.

**Table 2:**
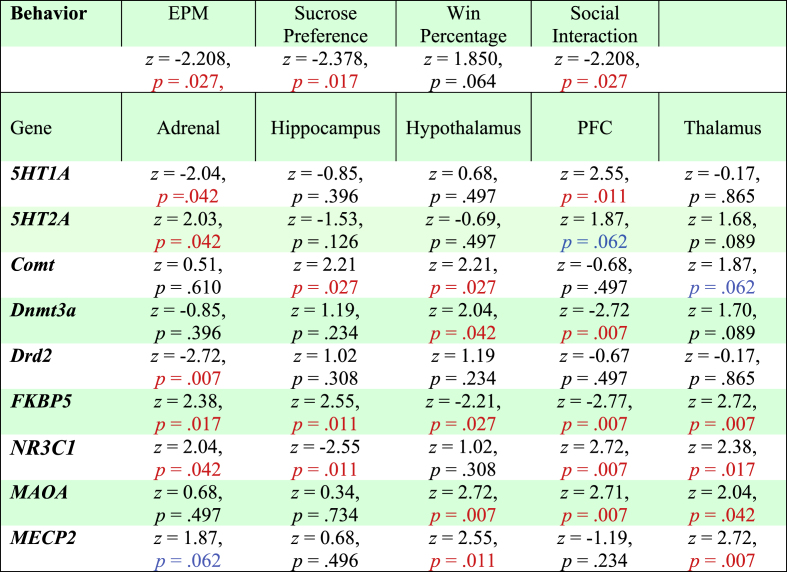
Results from Mann Whitney *U* test's examining behavioral differences and changes in gene expression between control sires and those exposed to combat-related trauma. Red text indicates significant effects.

A one-way repeated measures ANOVA for corticosterone levels, identified higher corticosterone levels in combat-related trauma sires, (F_1.2,13.56_ = 7.388, *p* = .034), that was not affected by time (F_1.2,13.56_ = 0.39, *p* = .894; Fig. 1G).

### 3.2 Offspring behavior prior to the adolescent challenge

**Fig. 2:**
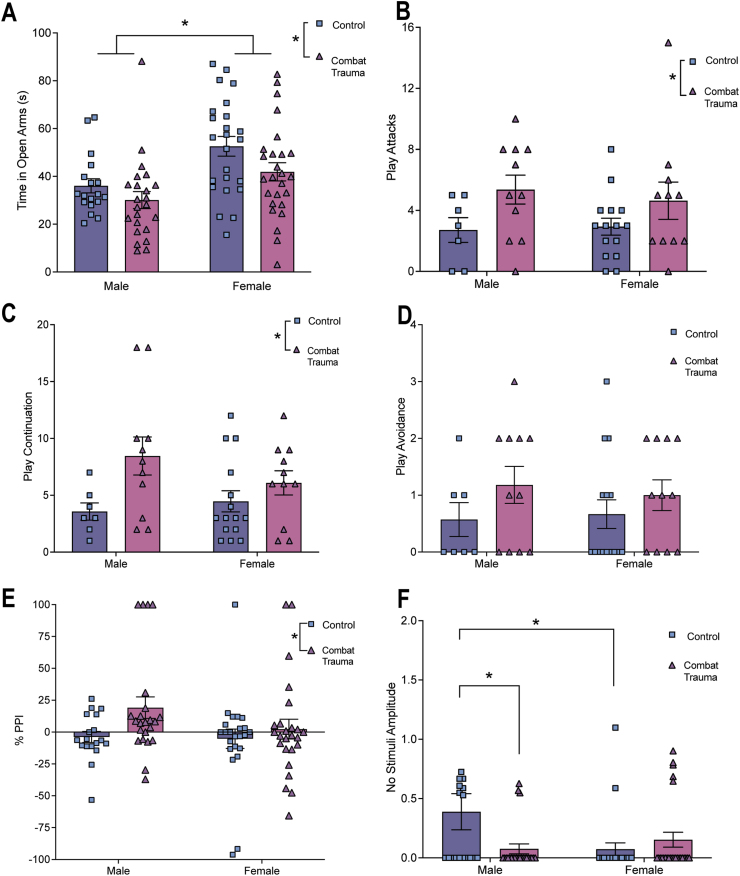
Bar graphs displaying the behaviour results of offspring prior to the stress challenge in adolescence. Means ± SEM. Significance indicated by ∗; *p's* < 0.05. A) Time spent in the open arms of the elevated plus maze; B) Play attacks in the social interaction test; C) Play continuation in the social interaction test, with a main effect of paternal group; D) Avoidant behaviours in the social interaction test; E) PPI% from the acoustic startle test; and F) Displays no stimuli amplitude from the acoustic startle test.

Statistical results for all two-way ANOVAs (and post-hoc analyses where appropriate) for offspring behavior prior to the adolescent challenge can be found in Table 3. Overall, we found that rats born to sires in the combat trauma group, and males, spent significantly less time in the open arms of the EPM (Fig. 2A). In addition, offspring born to sires exposed to the combat trauma paradigm exhibited increased sociability, whereby they initiated more attacks on the naïve rats, were attacked more often by the naïve rats, and exhibited increased play continuation behaviors compared to control. There was no effect of paternal group on play avoidance behaviors (Fig. 2B–D).

**Table 3:**
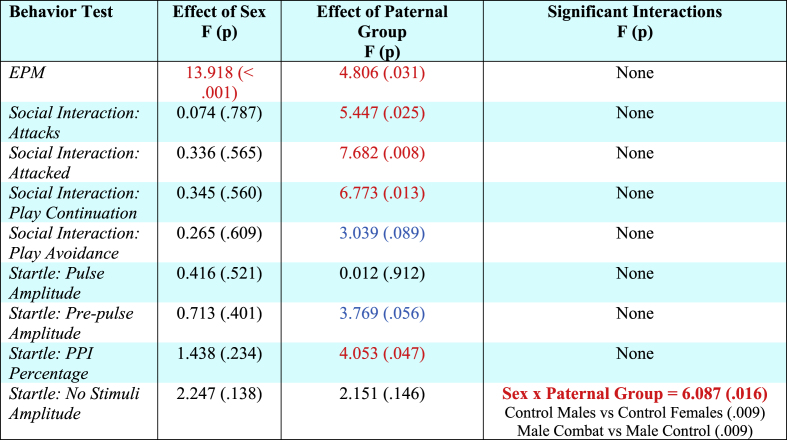
Results from the two-way ANOVAs for the offspring behavioral tests performed prior to the adolescent stress challenge. Red text denotes significant effects, while blue text denotes trends towards significance.

With respect to startle behaviors, paternal trauma had no effect on pulse amplitude and pre-pulse amplitude. However, offspring from the combat trauma group had higher PPI percentage values than offspring born to control sires (Fig. 2E). Further, there was a significant sex by paternal group interaction in the no stimuli amplitude, driven by offspring born to control sires, whereby males had higher values than females. For male offspring, those born to sires in the combat trauma group had lower values, indicating an increase in un-prompted freezing behaviour (Fig. 2F).

### 3.3 Offspring behavioral results following the adolescent stress exposure

**Fig. 3:**
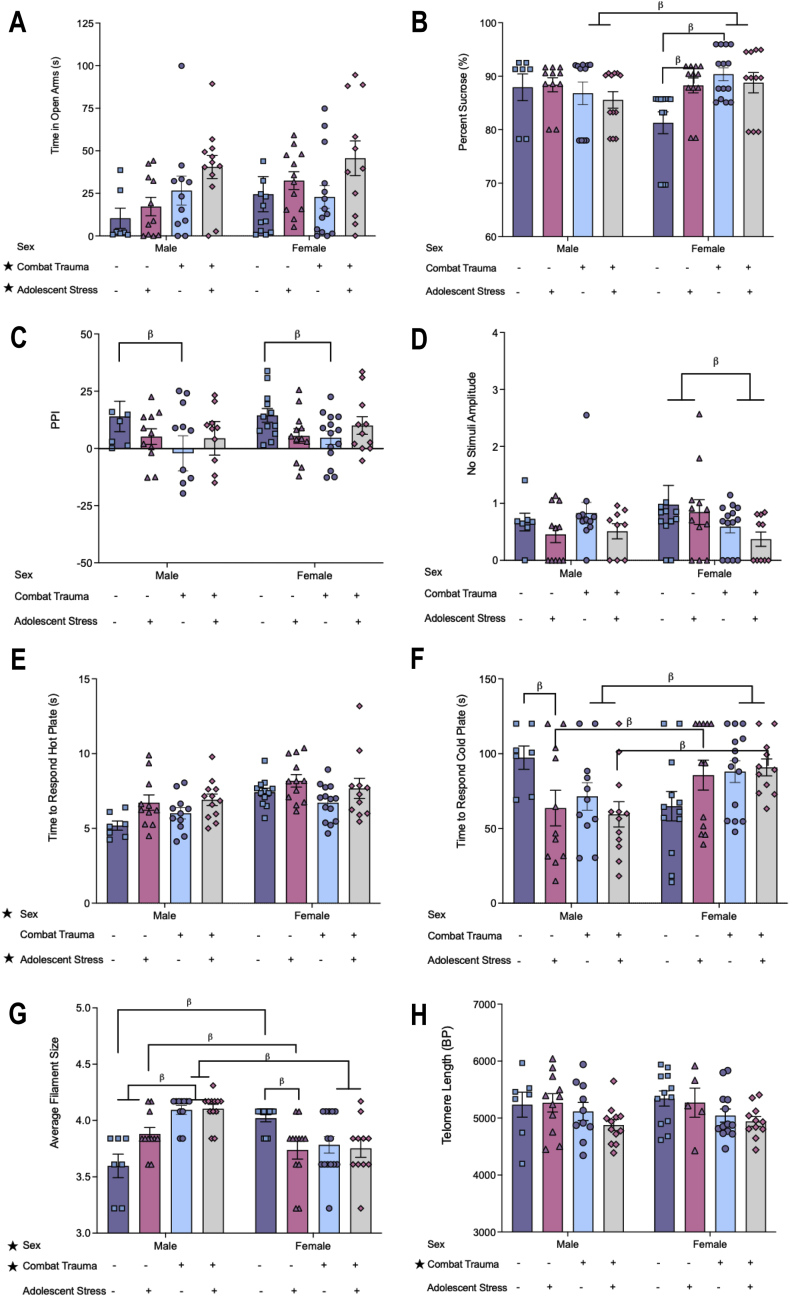
Bar graphs displaying the behaviour results for offspring born to combat trauma or control sires, following exposure to the stress challenge in adolescence. Graphs depict means ± SEM. ∗ indicates a significant main effect, β indicates a significant interaction; *p's* < 0.05. A) Displays time spent in the open arms of the elevated plus maze; B) Percent sucrose intake in the sucrose preference test; C) PPI% from the acoustic startle test; D) Amplitude for responses to no-stimuli in the acoustic startle test; E) Time to respond on the hot plate; F) Time to respond on the cold plate; G) Average filament size on the von Frey task; and H) Average ear notch telomere length.

Statistical results for all three-way ANOVAs (and post-hoc analyses where appropriate) for offspring behavior following the adolescent challenge can be found in Table 4. In summary, time spent in the open arms was increased in offspring born to sires in the combat trauma group as well as for offspring exposed to the predator stressor, indicating a reduction in anxiety-like behaviors (Fig. 3A). The sucrose preference test demonstrated that females consumed a greater percentage of sucrose than males when they were born to sires in the combat trauma group. Further, a paternal group by adolescent challenge interaction was observed, where offspring born to fathers in the combat trauma group consumed less sucrose when they also experienced the adolescent stressor. Conversely, offspring born to control sires, consumed more sucrose when presented with the adolescent stressor. (Fig. 3B).

**Table 4:**
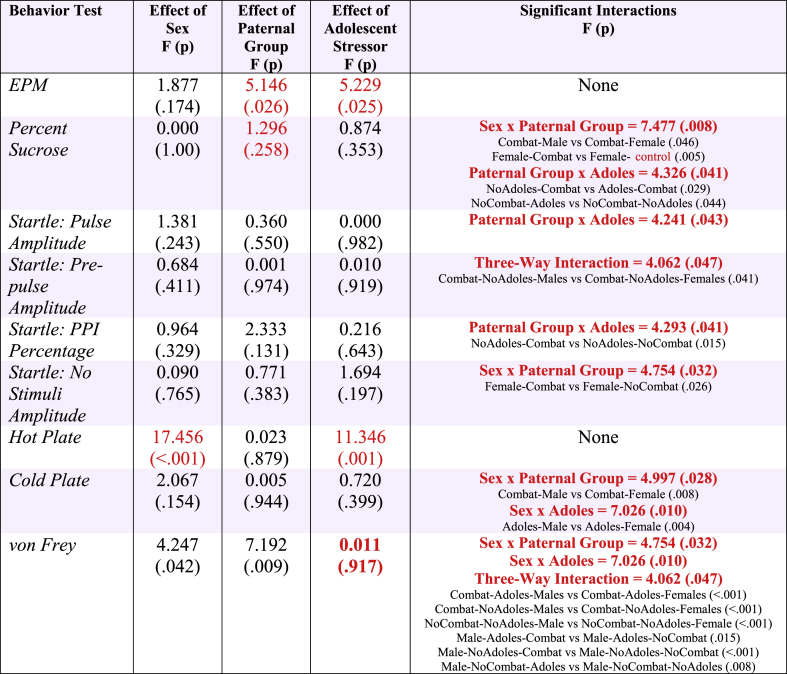
Results from the three-way ANOVAs for the offspring behavioral tests performed after the adolescent stress challenge. Red text denotes significant effects.

We identified a three-way interaction for pre-pulse amplitude, driven by offspring in the combat trauma group, whereby males who did not experience the adolescent stressor paradigm had higher values than females in this group. Further, a paternal group by adolescent challenge interaction was observed for PPI percentage where offspring born to sires from the combat trauma group exhibited reductions in PPI if they did not experience stress in adolescence (Fig. 3C). Lastly, with respect to the no stimuli amplitude, where female offspring born to sires in the combat trauma group had lower values than offspring born to control sires (Fig. 3D) suggesting an increase in un-prompted freezing behaviour.

Finally, we examined thermal and mechanical nociception. On the hot plate, females and offspring exposed to stress in adolescence exhibited reduced sensitivity to noxious heat. On the cold plate test female offspring born to combat trauma fathers also exhibited reductions in thermal sensitivity when compared to males born to combat trauma fathers. Further, when paternal combat exposure was combined with the adolescent stressor, females exhibited further reductions in sensitivity compared to their male counterparts. Additionally for male offspring, exposure to any of the three conditions (paternal trauma; adolescent stress; both paternal trauma and adolescent stress) increased thermal sensitivity to cold stimuli (Fig. 3F). On the von Frey task, female offspring exposed to any of the three conditions (paternal trauma; adolescent stress; both paternal trauma and adolescent stress) exhibited an increase in mechanical sensitivity (Fig. 3G). Conversely, for male offspring, exposure to any of the three conditions reduced mechanical sensitivity, with the effect being most significant when they were born to a sire in the combat trauma group.

### 3.4 Paternal and offspring telomere length and gene expression

**Fig. 4:**
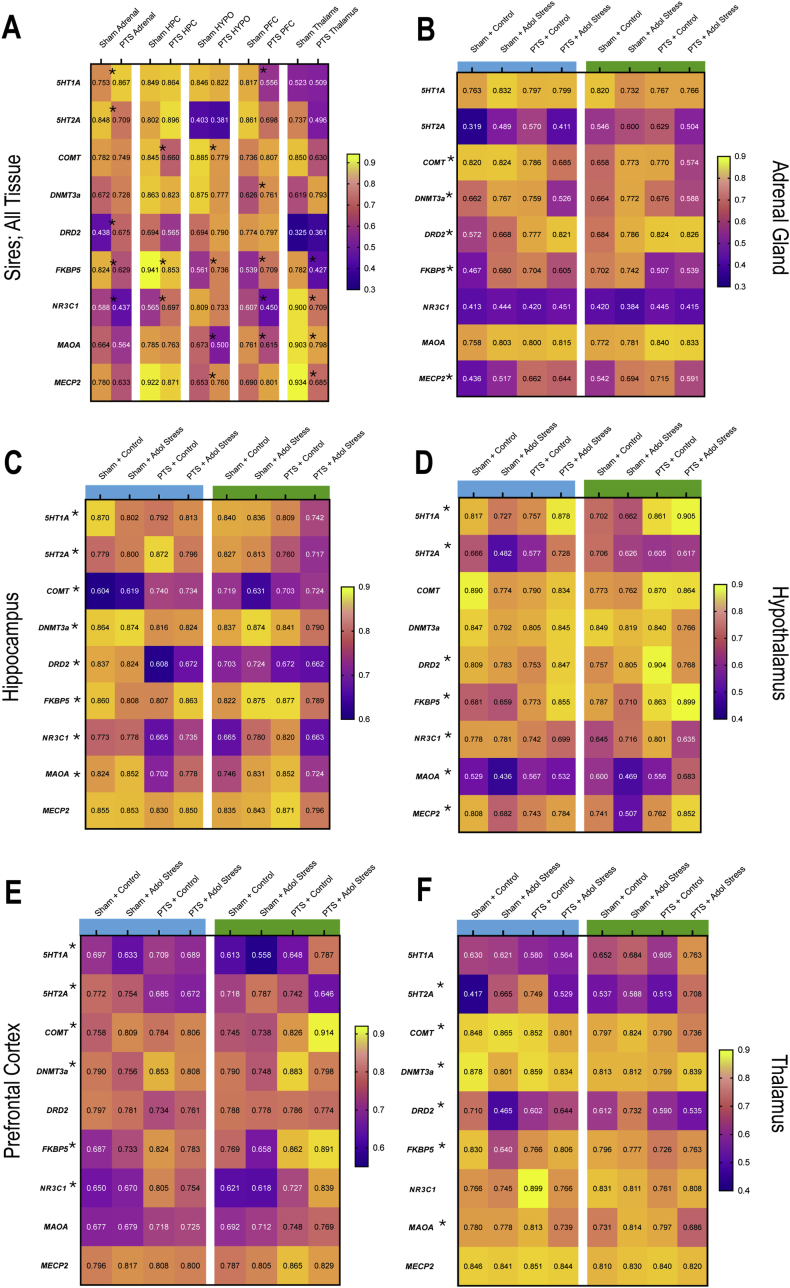
Heat maps illustrating changes in gene expression. A) For control sires and sires exposed to the combat trauma paradigm. B-F) Changes in gene expression in the adrenal gland, hippocampus, hypothalamus, prefrontal cortex, and thalamus, for offspring from each experimental condition. Blue bars denote male offspring and green bars denote female offspring. The ∗ indicate a significant main effect or significant interaction, *p* < .05. *n* = 6 for each box within the heat map.

Telomere length was measured in sires and their offspring. There were no significant differences in telomere length for sires, *z* = −0.426, *p* = .670 (average base pairs: combat trauma sires, 5151.19 ± 220; control sires, 4924.74 ± 279, data not shown). Average telomere length was reduced in offspring born to sires exposed to the combat trauma group, (F_1,81_ = 7.296, *p* = .009; Fig. 3H). Table 2 and Fig. 4A contain the statistical results and heatmaps for the gene expression data obtained for the sires. In brief, the combat trauma paradigm modified *FKBP5* expression in all tissues examined. Similarly, *NR3C1* expression was modified in the combat trauma group for all tissues except the hypothalamus. Expression of the epigenetic regulators, *MECP2* and *Dnmt3a*, were increased in the hypothalamus and PFC, respectively. Within the thalamus, all genes modified by the combat trauma paradigm were down regulated (*FKBP5, NR3C1, MAOA,* and *MECP2*). Exposure to the combat trauma paradigm only modified expression of the *Drd2* gene in the adrenal glands.

The statistical results for the three-way ANOVAs examining gene expression changes in the offspring can be found in Table 5. Fig. 4B–F contain the heatmaps that visually display the three-way ANOVA results. In summary, the offspring PFC was most significantly affected by paternal trauma, with 6/9 genes (*5HT1A, 5HT2A, Comt, Dnmt3a, FKBP5, NR3C1*) exhibiting changes in response to the paternal experience. Gene expression changes in the hypothalamus and thalamus were primarily evident when paternal trauma was combined with the stress exposure in adolescence (5/9 genes from each tissue exhibited a significant paternal by adolescent interaction or a significant three-way interaction). Similar to their fathers, *Drd2* expression was significantly increased in the adrenal glands of offspring who were born to sires from the combat trauma group. Surprisingly, expression of only two genes, *Comt* in the hippocampus and *Dnmt3a* in the PFC, were modified in response to the adolescent stressor. There were also very few sex-dependent changes in gene expression with only adrenal and thalamic expression of *Comt* being lower in females compared to males. Sex interacted with paternal group to affect expression of *Comt* in the PFC, *5HT2A* in the hippocampus, and *FKBP5* in the adrenal glands, where *FKBP5* was reduced in female offspring born to combat trauma sires but increased in male offspring in the same group.

**Table 5:**
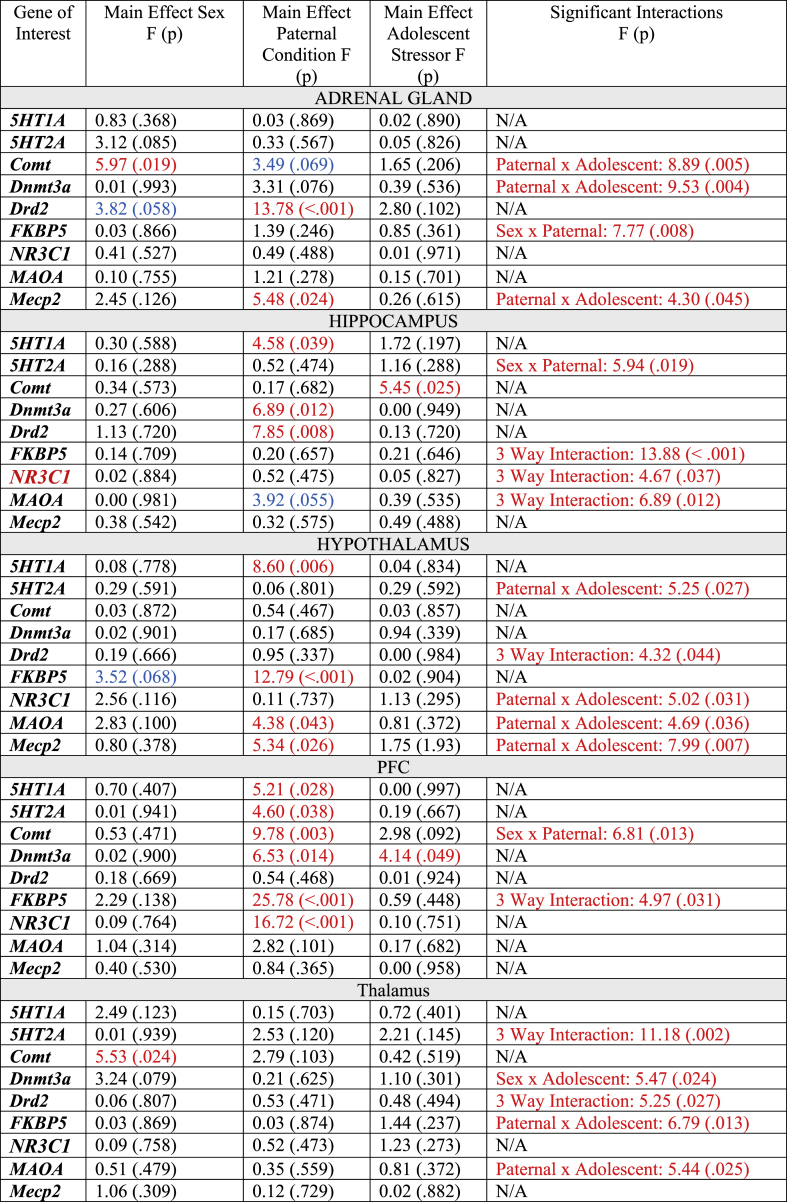
Statistical output for the 3-Way ANOVAs for gene expression changes in the adrenal gland, hippocampus, hypothalamus, PFC, and thalamus of offspring born to control or trauma-induced sires as well as exposure to a stressor or control condition in adolescence. Red text indicates significant effects.

## 4 Discussion

PTS and concussion in military Veterans are significant health concerns that impose substantial risk to the health and wellbeing of their children. The trauma and subsequent PTS associated with combat is often induced by a variety of stimuli that manifest through both physical and psychological processes. Of critical importance, the physiological consequences of PTS persist, long after the exposures have ceased. As such, individuals experiencing PTS may inadvertently modify development and functioning of their offspring through epigenetic changes induced by the initial trauma and ongoing manifestation. We therefore examined the effects of paternal combat-related trauma on offspring behaviour and gene expression associated with HPA-axis functioning. The trauma protocol produced persistent changes in sire anxiety, anhedonia, and sociability, as well as elevated levels of corticosterone and changes to gene expression. These paternal experiences prior to conception changed offspring behaviour and gene expression but did not significantly modify their offspring's response to a significant stressor in adolescence.

The sire trauma paradigm aimed to mimic the factors related to combat-induced PTS, including mTBIs/concussions as well as stressful experiences that affect multiple sensory modalities (olfactory – fox urine; visual – hawk predator exposure; physical – restraint tube; auditory – acoustic startle) and in turn, result in allostatic overload. The trauma protocol increased circulating corticosterone levels, and 30 days following the final stress exposure, sires exhibited increased anxiety-like behaviour, consumed higher amounts of sucrose, and spent more time engaged in social interaction. While increased anxiety-like behaviours are typical in animal models of PTS (Zoladz et al., 2012; Flandreau and Toth, 2018), the increased sucrose consumption and social interaction was surprising. However, previous studies have found that environmental enrichment and social interaction are capable of buffering against the onset of trauma-induced behavioural manifestations (Seetharaman et al., 2016; Fox et al., 2006) and it is therefore possible that the 4 day mating period with a control female served a similar function for sires in the combat trauma group. Alternatively, there is a strong link between substance abuse and PTS in Veterans (Bowe and Rosenheck, 2015), suggesting that the increase in sucrose consumption and social interaction exhibited by these sires may have represented an increase in reward seeking behaviour following their exposure to the trauma. We also found that sires exhibited changes in gene expression consistent with PTS literature (Baghaei et al., 2024; Liu et al., 2023). For example, expression of *FKBP5,* a glucocorticoid receptor co-chaperone that helps regulate glucocorticoid receptor responsivity, and *NR3C1*, were modified in almost all tissues examined suggesting that the trauma paradigm significantly disrupted functioning of the stress response (Yehuda et al., 2016; Hawn et al., 2019). Sires exposed to trauma also exhibited changes in gene expression related to the dopaminergic and serotonergic systems (*5HT1A, 5HT2A, Comt, Drd2, MAOA*) and epigenetic regulators (*Dnmt3a, Mecp2*). Of importance, although the sires did not interact with their offspring in any capacity, many of the behavioural and transcriptomic changes associated with exposure to the trauma paradigm were transmitted to their offspring.

Early in life, offspring born to sires in the trauma group exhibited increases in anxiety-like behaviour, social engagement, and PPI. PPI is a reduction in the startle response when a weak, non-startling stimulus precedes an intense sound. A reduction in PPI is thought to reflect an inability or dysfunction in one's capacity to sustain attention and/or filter sensory information, and has been reported in Veterans with PTS (Grillon et al., 1996). Although we found an increase in offspring PPI for those born to sires in the trauma group when tested early in life (p25), testing in late adolescence (∼p50) identified reductions in PPI for these offspring. As some researchers have suggested that PTS is an overgeneralisation of the stress response to non-fearful stimuli and this overgeneralisation leads to a breakdown in inhibitory processes (Sutker et al., 1995; Echiverri-Cohen et al., 2016), it is possible that offspring born to trauma exposed sires have inherited this hyper-responsive stress response. Moreover, while the mechanisms responsible for PPI have not been fully defined, evidence does suggest that dopamine and the *Drd2* gene play a fundamental role (Wan and Swerdlow, 1993). Social interaction and play behaviours have also been linked to the dopaminergic system and *Drd2*, as play is a highly rewarding behaviour (Sgro and Mychasiuk, 2020). Similar to their fathers, offspring born to sires in the trauma group exhibited increased play initiation and play continuation behaviour, similarly suggesting an increased motivation for the rewarding aspects of play. At the time of euthanasia, offspring born to sires in the trauma group exhibited changes to expression of *Drd2* in the adrenal glands, hippocampus, hypothalamus, and thalamus. It is interesting to note, that the beneficial effects of paroxetine treatment (a selective serotonin reuptake inhibitor) on social functioning for Veterans with PTS are believed to be mediated by the *Drd2* gene (Lawford et al., 2003).

Drd2 receptors in the adrenal glands are particularly important for the modulation and secretion of catecholamines such as epinephrine and norepinephrine, playing a significant role in the fight-or-flight response (Pivonello et al., 2004). We identified an increase in *Drd2* expression in the adrenal glands of both sires exposed to the trauma paradigm as well as their offspring. This could be a compensatory mechanism to assist in the modulation and suppression of chronic allostasis induced by the trauma paradigm that was passed to the next generation. In many human studies, the A1 DRD2 allele, which results in reduced Drd2 receptor density, is associated with increased PTS prevalence (Liu et al., 2023; Zhang et al., 2023). Offspring born to sires exposed to the trauma paradigm exhibited reductions in *Drd2* expression within the hippocampus and thalamus (male offspring also experienced reductions in *Drd2* expression in the hypothalamus), suggesting they may be at risk for the development of PTS and its associated comorbidities.

With respect to offspring outcomes in late adolescence, we hypothesised that the paternal trauma experience would modify their response to the hawk exposure. Within the maternal trauma literature, the effects of adversity tend to be cumulative; when maternal trauma is combined with offspring adversity, the effects are exacerbated (Beveridge et al., 2018, 2020; Sgro et al., 2025). Despite numerous changes to gene expression within the HPA axis, paternal trauma did not exacerbate the offspring's response to this additional stressor. Some researchers have hypothesised that paternal stress prior to conception alters the timing of developmental cascades involved in the stress signal, shifting the neurobiological, physiological, and behavioural response of offspring to stressful environments (Duffy et al., 2021). Given this, paternal stress may result in small changes that produce significant outcomes over time. As such, we may have identified a larger response if we examined offspring at a later age, or in response to repeated stress exposures in adolescence.

Behavioural testing in late adolescence did however reveal numerous sex differences, where male and female offspring born to sires in the trauma group exhibited opposing outcomes, suggesting that paternal inheritance of trauma manifests in a sexually dimorphic manner. This is not a surprising outcome, given that human studies examining paternal trauma have identified sex differences in the intergenerational transmission of symptomology. For example, one study of prisoners of war found that although sons were at greater overall risk for the development of PTS, paternal PTS was positively linked to their daughter's, but not their son's symptoms (Zerach and Solomon, 2018). Of importance, cold and mechanical nociceptive sensitivity were two of the behavioural tasks that exhibited an interaction between sex and paternal trauma with respect to offspring outcomes. Although male and female offspring born to sires in the trauma group exhibited opposite changes in nociceptive sensitivity, both presented with abnormalities in nociception that are associated with increased risk for the development of chronic pain (Seminowicz et al., 2009; Salberg et al., 2023). While literature clearly indicates that both Veterans and civilians living with PTS have significantly higher prevalence rates of chronic pain than the general population (Brennstuhl et al., 2015; Fishbain et al., 2017; Scioli-Slater et al., 2015), this study suggests that fathers may pass this risk onto their offspring. The reductions in telomere length provide further support to this hypothesis as shortened telomeres have been associated with numerous disease states and accelerated aging (von Zglinicki, 2002; Hassett et al., 2012; Entringer et al., 2012; Blasco, 2005).

PTS-induced changes in the heritable expression of specific genes related to pain perception and neurotransmission may play a significant role in the intergenerational risk for, and development of, chronic pain conditions (Denk and McMahon, 2012; Edwards et al., 2016; Woolf, 2010). For example, genetic variants of the *Comt* gene (Andersen and Skorpen, 2009), and changes to expression of *Comt* (Christensen et al., 2021) have been linked to acute pain sensitivity as well as susceptibility to the development of chronic pain conditions. Interestingly, the majority of baseline sex differences in gene expression we identified, were related to *Comt*. It is therefore possible that the significant variation in nociceptive sensitivity identified in male and female offspring born to sires in the trauma group occurred because the inheritance of risk associated with the paternal trauma exposure acted upon nociceptive circuits that were developing in a sexually dimorphic manner.

*Comt* also plays a significant modulatory role in the dopamine system as it inactivates catecholamines; whereby reductions in *Comt* are associated with higher levels of dopamine in the brain (Liu et al., 2023). Given that dopamine plays a key role in the formation, consolidation, and extinction of fear memories, dysregulated dopamine neurotransmission has been linked to the development and maintenance of PTS (Liu et al., 2023). Within this study, sires exposed to trauma had reductions in *Comt* in the hippocampus and hypothalamus possibly resulting in increased dopamine and a greater propensity to form and activate fear memories. Conversely, offspring born to trauma sires had increased *Comt* expression in the hippocampus and PFC, which may reflect a compensatory mechanism to offset the mismatch in environment between the period prior to conception and postnatal life, that has occurred given the lack of continuous random stress, that the paternally induced changes had primed the brain for (Hanson and Gluckman, 2008).

Paternal trauma exposure significantly modified gene expression of *Dnmt3a* and *Mecp2* in sires and offspring. As *Dnmt3a* and *Mecp2* are involved in regulating the methylation status of other genes, they strongly influence the neurological response to environmental stimuli by modulating processes such as dendritic arborization and synaptic plasticity (Feng et al., 2010). The changes in expression of these two genes in both generations highlights the capacity for intergenerational inheritance of epigenetic risk and resiliency. In addition, war and combat PTSD symptom severity and symptom presentation has been linked to variations in the genetic composition of the *MAOA* allele, as well as, to increases in *MAOA* methylation; however, this relationship is restricted to males (Ziegler et al., 2018; Kravic et al., 2019). In line with this, we identified reduced *MAOA* expression in the hippocampus, PFC, and thalamus of sires exposed to the trauma paradigm. *MAOA* is also believed to play a role in the relationship between childhood trauma and adult mental health, including aggression and PTS (Frazzetto et al., 2007; Montalvo-Ortiz et al., 2013). We found that being born to a father in the paternal trauma group resulted in reduced expression of *MAOA* in the hippocampus of male offspring, but increased expression of *MAOA* in the hippocampus and thalamus of female offspring, providing further evidence for sex differences in *MAOA*-related susceptibility to negative environmental conditions.

Two of the most explored genes with respect to combat and war related PTS are *FKBP5* and *GR* (a.k.a. *NR3C1*). Combat Veterans with PTS have lower methylation levels in the promoter region of *NR3C1-1F* (Yehuda et al., 2015) suggesting dysregulation of the negative feedback inhibition loop within the HPA axis (Yehuda et al., 2015). Moreover, these effects were heritable; *FKBP5* methylation was reduced in the offspring of Holocaust survivors (Bierer et al., 2020), paternal PTSD was associated with greater methylation of the *NR3C1* gene in blood samples obtained from offspring (Yehuda et al., 2014), and PTS-induced alterations to DNA methylation of *NR3C1* and *FKBP5* in paternal sperm were linked to mental health diagnoses in offspring (Mehta et al., 2019). In line with this, we found alterations to *NR3C1* expression in the PFC and hippocampus of offspring born to sires in the trauma group. We also linked changes in expression of *FKBP5* from all tissues examined to combat exposure in the sires. These changes in offspring *FKBP5* expression provide further evidence that the intergenerational transmission of risk for chronic pain can be mediated by fathers, as a previous study has shown that for individuals who experience increased chronic stress, changes in *FKBP5* levels predict musculoskeletal pain severity (Ulirsch et al., 2014).

In summary, the three-week stress paradigm designed to mimic combat-specific trauma in military personnel, significantly modified sire behaviour, cortisol levels, and gene expression within the HPA-axis and related brain structures. As a consequence, offspring born to fathers who experienced this trauma exhibited changes to their behaviour and gene expression, which suggests intergenerational risk for the development of PTS and associated comorbidities. Although the offspring's response to the adolescent stressor was not affected by paternal trauma, offspring did exhibit reduced anxiety-like behaviour, PPI, and telomere length, as well as changes in sensitivity to nociceptive stimuli and gene expression throughout the HPA-axis. In line with other rodent models of PTS, one could question whether the effects we identified in the progeny were based upon the stress to the fathers, or the manifestation of the exposure, given that we only provided three days of rest between trauma and mating. Future studies could use sires that undergo the trauma paradigm but are not mated for an extended period. This would potentially resolve this inconsistency in the literature; however, the effects of advanced paternal age would confound the outcomes (Hehar et al., 2016; Smith et al., 2013). It is also important to highlight that although intergenerational transmission from fathers to offspring may be largely driven by epigenetic processes, paternal experiences prior to conception can influence maternal care and investment in offspring (Hehar and Mychasiuk, 2015b; Mashoodh et al., 2012), and while not a factor in this study, paternal behaviours and parenting styles following birth also influence offspring outcomes in species such as humans where co-parenting is common (Freeman et al., 2025; Hehar and Mychasiuk, 2015b). Lastly, as many studies examining the effects of paternal experience have noted greater change in the F2 generation (Bygren et al., 2001; Frans et al., 2011), future studies could monitor the subsequent generation to determine if similar results are identified with this model of combat-induced PTS. Given that epigenetic changes are modifiable processes, this model of paternally-induced intergenerational transmission could be used to explore the efficacy of epi-drugs or epi-diets to ameliorate some risk imparted upon offspring by Veteran fathers living with PTS (Farani et al., 2023). Therapies targeting the methylation patterns of specific genes may be able to compensate for the paternal trauma-induced changes to epigenetic processes.

## Data availability statement

All raw data is available at the open-source framework repository – https://osf.io/vbg5q/?view_only=9a03ce046fd744bfb4899eae965bb2fe.

## Funding and acknowledgements

The authors would like to thank and acknowledge Australia's 10.13039/501100000925National Health and Medical Research Council for their financial contribution (AP1173565 to RM).

## CRediT authorship contribution statement

**Glenn R. Yamakawa:** Data curation, Formal analysis, Investigation, Methodology, Writing – original draft, Writing – review & editing. **James Freeman:** Conceptualization, Data curation, Methodology, Writing – original draft, Writing – review & editing. **Sydney Harris:** Data curation, Formal analysis, Methodology, Writing – original draft, Writing – review & editing. **Marissa Sgro:** Data curation, Formal analysis, Methodology, Writing – original draft, Writing – review & editing. **Elaina Vlassopoulos:** Data curation, Formal analysis, Methodology, Writing – original draft, Writing – review & editing. **Crystal N. Li:** Data curation, Formal analysis, Methodology, Writing – original draft. **Josep Roman-Juan:** Data curation, Formal analysis, Writing – original draft, Writing – review & editing. **Melanie Noel:** Conceptualization, Methodology, Writing – original draft, Writing – review & editing. **Sabrina Salberg:** Data curation, Formal analysis, Investigation, Methodology, Project administration, Supervision, Validation, Writing – original draft, Writing – review & editing. **Richelle Mychasiuk:** Conceptualization, Data curation, Formal analysis, Funding acquisition, Investigation, Methodology, Project administration, Resources, Software, Supervision, Validation, Visualization, Writing – original draft, Writing – review & editing.

## Declaration of competing interest

The authors declare the following financial interests/personal relationships which may be considered as potential competing interests: Richelle Mychasiuk reports financial support was provided by 10.13039/501100000925National Health and Medical Research Council. If there are other authors, they declare that they have no known competing financial interests or personal relationships that could have appeared to influence the work reported in this paper.

## Data Availability

The raw data isavailable at the open source framework repository: https://osf.io/vbg5q/overview?view_only=9a03ce046fd744bfb4899eae965bb2fe
